# Accelerated long‐term forgetting over three months in asymptomatic APOE ɛ4 carriers

**DOI:** 10.1002/acn3.51245

**Published:** 2020-12-25

**Authors:** Adrià Tort‐Merino, Matti Laine, Natalia Valech, Jaume Olives, María León, Mirian Ecay‐Torres, Ainara Estanga, Pablo Martínez‐Lage, Juan Fortea, Raquel Sánchez‐Valle, Lorena Rami, Antoni Rodríguez‐Fornells

**Affiliations:** ^1^ Alzheimer’s Disease and other Cognitive Disorders Unit Neurology Service, Hospital Clínic of Barcelona Barcelona Spain; ^2^ Institute of Neurosciences University of Barcelona Barcelona Spain; ^3^ Department of Psychology Åbo Akademi University Turku Finland; ^4^ Centro de Investigación y Terapias Avanzadas, Neurología CITA‐Alzheimer Foundation San Sebastián Spain; ^5^ Memory Unit, Department of Neurology Hospital de la Santa Creu i Sant Pau and Institute of Biomedical Research Barcelona Spain; ^6^ August Pi i Sunyer Biomedical Research Institute (IDIBAPS) Barcelona Spain; ^7^ Cognition and Brain Plasticity Group Bellvitge Biomedical Research Institute‐IDIBELL Hospitalet de Llobregat Spain; ^8^ Department of Cognition, Development and Education Psychology University of Barcelona Hospitalet de Llobregat Spain; ^9^ Catalan Institution for Research and Advanced Studies (ICREA) Barcelona Spain

## Abstract

Accelerated long‐term forgetting (ALF) refers to a rapid loss of information over days or weeks despite normal acquisition/encoding. Notwithstanding its potential relevance as a presymptomatic marker of cognitive dysfunction, no study has addressed the relationship between ALF and Alzheimer’s disease (AD) biomarkers. We examined ALF in APOE ɛ4 carriers versus noncarriers, and its relationships with AD cerebrospinal fluid (CSF) biomarkers. We found ALF over three months in APOE ɛ4 carriers (*F*(1,19) = 5.60; *P *< 0.05; Cohen’s *d* = 1.08), and this performance was associated with abnormal levels of the CSF Aβ_42_/ptau ratio (r = −.614; *P *< 0.01). Our findings indicate that ALF is detectable in at‐risk individuals, and that there is a relationship between ALF and the pathophysiological processes underlying AD.

## Introduction

Accelerated long‐term forgetting (ALF) has been defined as a rapid loss of information over days or weeks despite normal acquisition and encoding. Recently, the potential usefulness of ALF in the early detection of subtle cognitive difficulties in asymptomatic stages of familial^1^ and sporadic^2^, ^3^ Alzheimer’s disease (AD) has attracted particular attention. Two recent studies showed larger ALF in autosomal dominant disease mutation carriers^1^ and asymptomatic individuals with increased genetic risk (APOE ɛ4 carriers)^3^ over a 1‐week retention period.

Despite the potential relevance of ALF as a presymptomatic marker of subtle cognitive dysfunction, no study has yet addressed the relationship between ALF and AD biomarkers, or even evaluated ALF over periods beyond one week. To fill this gap, we employed an effortful and cognitively demanding associative memory task to explore ALF during a 6‐month follow‐up in cognitively healthy carriers versus noncarriers of the APOE ɛ4 haplotype. Moreover we examined the relationship between ALF and AD cerebrospinal fluid (CSF) biomarker levels. The possible findings derived from the present work would be of particular relevance for AD prevention trials, as well as for the assessment and monitoring of cognitively unimpaired populations at risk of AD.

## Participants and methods

The present participants represent a sub‐sample of Tort‐Merino et al.,^2^ and thus the methods description follows that paper. Among all the participants included in this previous work, 11 subjects were APOE ɛ4 carriers (heterozygous for ɛ3 and ɛ4). In the present study, these 11 carriers were age‐, sex‐, and education‐matched with 11 noncarriers (homozygous for ɛ3). The study was not pre‐registered, and thus the present analyses are exploratory. All subjects underwent APOE genotyping, a lumbar puncture to determine CSF amyloid‐ß (Aβ_42_), total tau (tau), and phosphorylated tau (ptau) levels, a standard neuropsychological assessment to ensure that they were cognitively normal, and the cognitively demanding Ancient Farming Equipment Test (AFE‐T) to tap learning and long‐term retention at 1 week, 3 and 6 months. Participants were blind to APOE status and CSF results. The mean time lapse between the lumbar puncture and the AFE‐T assessment was 2.09 (1.4) years. The neuropsychological battery encompassed four cognitive domains (memory, language, perception, and executive functions; see Tort‐Merino et al.^2^ for tests details), and it included the Cognitive Reserve Questionnaire (CRQ) for assessing cognitive reserve^4^ and the Subjective Cognitive Decline Questionnaire (SCD‐Q)^5^ for measuring cognitive concerns.

The AFE‐T calls for learning novel object/name pairs: it contains 24 black‐and‐white images of unfamiliar ancient farming equipment taken from the AFE paradigm^6^ which are paired with a pseudoword. The test consists of two initial learning sessions administered on two consecutive days. Each learning session include seven learning runs (range of performance score 0‐24 points per run). The AFE‐T provides two final learning outcomes: (1) the free learning score (FLS) and (2) the cued learning score (CLS). Long‐term recall is examined one week, three months and six months after the initial learning phase, including a visual recognition task, free recall, and cued recall (participants are not told in advance that they will be reassessed). Forgetting rates for free and cued recall scores at 1 week, 3 months, and 6 months were obtained with comparisons to the initial learning phase. The forgetting rate was defined as one minus the ratio between each delayed score (free or cued) and the score obtained on the FLS (for free forgetting rates) or CLS (for cued forgetting rates) [e.g., 1‐(one‐week free recall score/ FLS), for one‐week free forgetting rate; and 1‐(one‐week cued recall score/ CLS), for 1‐week cued forgetting rate]. Recall rates at 3 months and 6 months were also compared with the 1‐week recall session. At the end of the 6‐month session, a verbal recognition task was given. Concerning the APOE carriers, one subject did not obtain the CLS and three subjects refused to complete the 6‐month assessment.

In order to mathematically model the forgetting functions, we followed previous recommendations.^7^, ^8^, ^9^ Forgetting curves are characterized by a curvilinear relation that shows a rapid initial decline of information that is followed by a slower and longer decay, thus showing that information is lost in a larger extent after initial encoding. Previous studies on forgetting have shown that these functions (power and logarithmic) are the most accurate ones to describe forgetting curves. Information decay with time in our sample was fitted better using a logarithmic function [(y = a–bln(time)] when compared to a power function. Thus, each data point (4‐time data points) for each subject and condition (free and cued recall conditions) was fitted using a nonlinear least‐squares regression. Fit parameters were calculated based on the residual sum of squares and showing the proportion of data variance accounted for (*R* ^2^). The *slope* (parameter *b*) and *intercept* (parameter *a*) were computed separately for each subject and condition. The *slope* captures the forgetting rate of encoded information while the *intercept* represents the estimated initial level of performance (immediately after the last learning run).

Demographical data, levels of CSF Aß_42,_ CSF tau, and CSF ptau were compared using Student *t*‐tests for independent samples and *Chi*‐square analyses when appropriate. Analysis of variance (ANOVA) with post‐hoc Bonferroni corrections and effect sizes expressed as Cohen’s *d* were conducted to compare the between‐group scores on the AFE‐T and the standard neuropsychological tests. Magnitude scale for Cohen’s *d* has been suggested as: *d* = 0.2, small effect size, *d* = 0.5, medium effect size and *d* = 0.8, large effect size.^10^ The AFE‐T scores included in the analyses were the two final learning scores (i.e., FLS and CLS) and the forgetting measures (raw scores and forgetting rates). Pearson’s bivariate correlations were used to assess the association between the forgetting measures (including the *slope* parameter from the modeled functions) with AD CSF biomarkers and subjective cognitive ratings.

## Results

### Group differences between APOE ɛ4 carriers and noncarriers

We observed no group differences in age, sex, years of education, or cognitive reserve. Regarding the AD biomarkers, there were no differences in CSF tau or p‐tau levels, but CSF Aβ_42_ levels were significantly lower (*t(20) = *3.98; *P *< 0.01) in the APOE ɛ4 carrier group (Table 1). There were no differences in any of the standard neuropsychological tests. Neither did the AFE‐T exhibit group differences in the learning scores (Table 1).

**Table 1:** Demographics, CSF levels, and AFE‐T results in the APOE ɛ4 carriers versus noncarriers

Parameters	APOE ɛ4 non‐carriers	APOE ɛ4 carriers	*F*	*P* ^1^
Demographics				
Gender (% women)	72.7%	72.7%	0.00^2^	1.00
Age	65.1 (6.5) [58‐75]	65.8 (6.9) [56‐77]	−0.23	0.814
Years of education	11.0 (3.3) [8‐18]	12.2 (4.3) [8‐20]	−0.78	0.446
CRQ	16.4 (3.9) [9‐22]	15.4 (5.5) [6‐22]	0.46	0.651
CSF levels				
Aβ_42_	873.9 (294.1) pg/ml	470.5 (163.1) pg/ml	3.98	0.001**
Tau	247.4 (98.7) pg/ml	307.1 (95.2) pg/ml	−1.44	0.165
Ptau	53.8 (14.8) pg/ml	59.1 (15.7) pg/ml	−0.81	0.423
AFE‐T				
Free learning score (FLS)	17.8 (6.2)	14.2 (6.8)	1.62	0.218
Cued learning score (CLS)	21.0 (3.2)	18.6 (4.8)	1.87	0.187
1‐week free recall	14.1 (6.5)	9.8 (7.4)	2.05	0.168
1‐week cued recall	18.2 (4.6)	15.1 (5.9)	1.86	0.188
1‐week FFR	0.15 (0.4)	0.41 (0.3)	2.54	0.127
1‐week CFR	0.14 (0.1)	0.21 (0.2)	1.21	0.285
3‐month free recall	7.1 (6.9)	3.4 (4.6)	2.18	0.155
3‐month cued recall	13.3 (5.5)	7.6 (6.4)	4.99	0.037*
3‐month FFR	0.59 (0.3)	0.82 (0.2)	3.76	0.067
3‐month CFR	0.37 (0.2)	0.62 (0.3)	5.60	0.029*
6‐month free recall	7.3 (6.2)	4.8 (5.4)	0.81	0.380
6‐month cued recall	13.1 (5.9)	10.2 (7.2)	0.89	0.358
6‐month FFR	0.58 (0.3)	0.74 (0.2)	1.61	0.222
6‐month CFR	0.38 (0.2)	0.48 (0.3)	0.67	0.423
1‐week to 3‐month FFR	0.51 (0.3)	0.75 (0.2)	3.52	0.076
1‐week to 3‐month CFR	0.27 (0.2)	0.57 (0.3)	8.70	0.008**
1‐week to 6‐month FFR	0.49 (0.3)	0.70 (0.2)	2.50	0.132
1‐week to 6‐month CFR	0.27 (0.2)	0.46 (0.3)	2.14	0.161
1‐week visual recognition	48.0 (0.0)	47.5 (1.5)	1.44	0.244
3‐month visual recognition	46.3 (1.8)	45.7 (1.6)	0.71	0.409
6‐month visual recognition	46.0 (1.5)	46.1 (0.8)	0.04	0.839
6‐month verbal recognition	21.9 (2.3)	22.4 (1.6)	0.26	0.616

Data are presented as means (SD; standard deviation) [range]. Key: CRQ, cognitive reserve questionnaire; CSF, cerebrospinal fluid; Aβ_42_, amyloid‐β isoform 42; Tau, total tau; ptau, phosphorylated tau; FFR, free forgetting rate; CFR, cued forgetting rate.

^1^ *p* values were determined by Student’s t‐test for demographics and CSF levels;

^2^ X^2^ statistic.

* *p* < 0.05

** *p* < 0.01.

#### Modeling of forgetting curves

**Figure 1:**
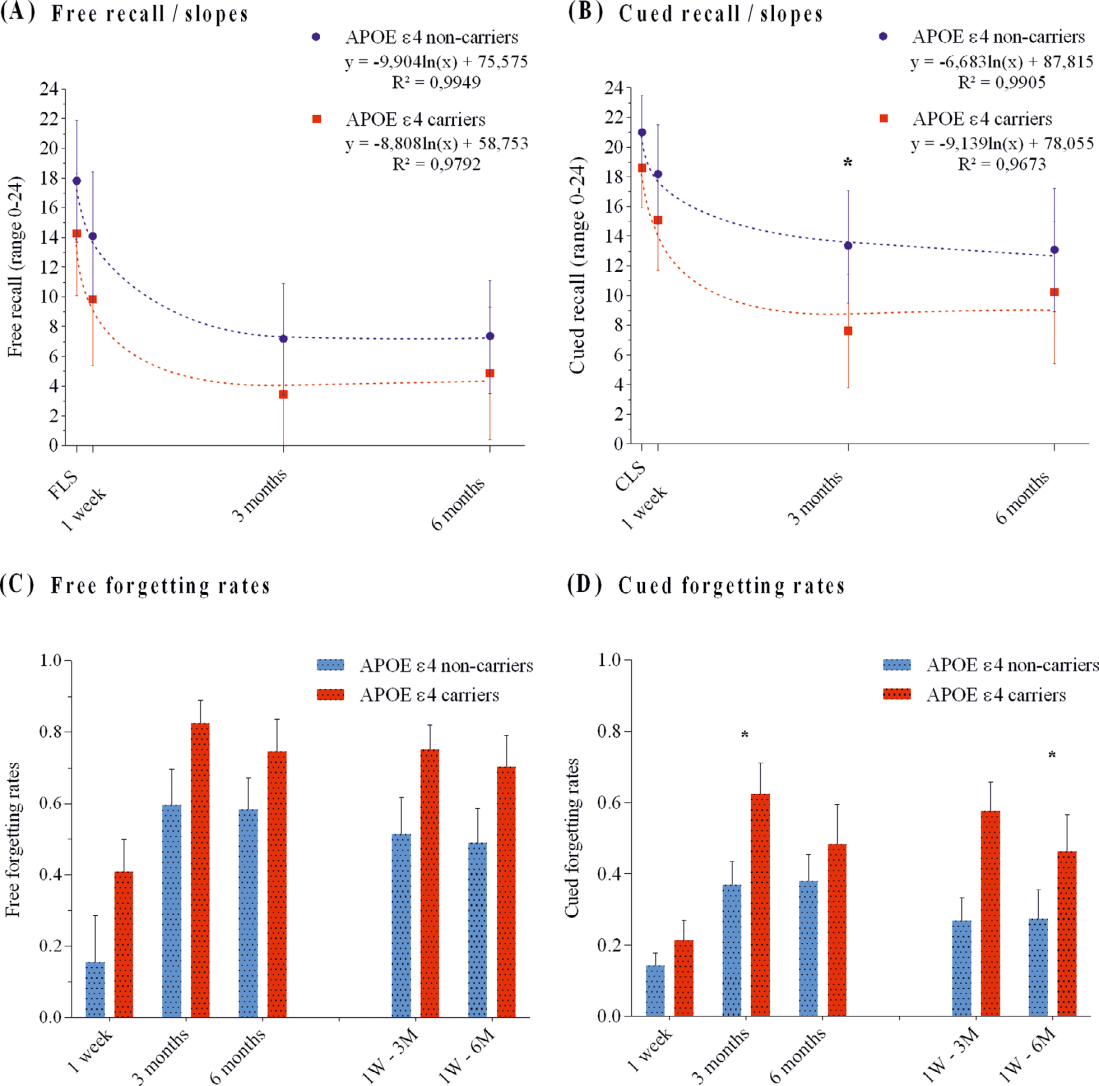
Panel A shows the free recall scores and the free slopes for APOE ɛ4 carriers and noncarriers. Panel B shows the cued recall scores and the cued slopes. Panels C and D show the free and cued forgetting rates, respectively. Key: FLS, Free learning score; CLS, Cued learning score; 1W‐3M, Forgetting rate between the 1‐week and 3‐month recall score; 1W‐6M, Forgetting rate between 1‐week and 6‐month recall. Error bars represent 95% CIs. *P < 0.05.

Figures 1A,B show the forgetting curves as well as the decaying information function modeled for the whole group. Importantly, the fit of the logarithmic function to the group data was nearly perfect in both groups, slightly better for the noncarriers (*R* ^2^ = 99% of variance explained) compared to carriers (*R* ^2^ = 97%). Particularly for cued recall (Figure 1B), the *slope* (forgetting rate parameter) was steeper for the carriers (*b* = −9.1) compared to noncarriers (*b* = −6.6). Importantly for the convergent validity of the different forgetting measures used (information decay mathematical model and forgetting rates at each time point, see results below), the 3‐month cued forgetting rate and the *cued slope* were strongly correlated (r = −.796; *P *< 0.01).

#### Forgetting rates at 1‐week, 3‐ and 6‐months

We computed forgetting rates as in previous studies of ALF^1^, ^3^ (see Figure 1C,D). At 3 months, cued recall score was significantly lower (*F*(1,19) = 4.99; *P *< 0.05; Cohen’s *d* = 0.99) in the carriers than in the noncarriers (Table 1; Figure 1B). Besides, the APOE ɛ4 carriers evidenced ALF in their 3‐month cued forgetting rate (*F*(1,19) = 5.60; *P *< 0.05; Cohen’s *d* = 1.08), as well as in their cued forgetting rate between the 1‐week and 3‐month recall (*F*(1,19) = 8.70; *P *< 0.01; Cohen’s *d* = 1.32; Post hoc power = 0.81) (Table 1; Figure 1D). No significant group differences were found on any of the free forgetting rates (Table 1; Figure 1C) or on the recognition scores (Table 1).

### Correlational analyses

**Figure 2:**
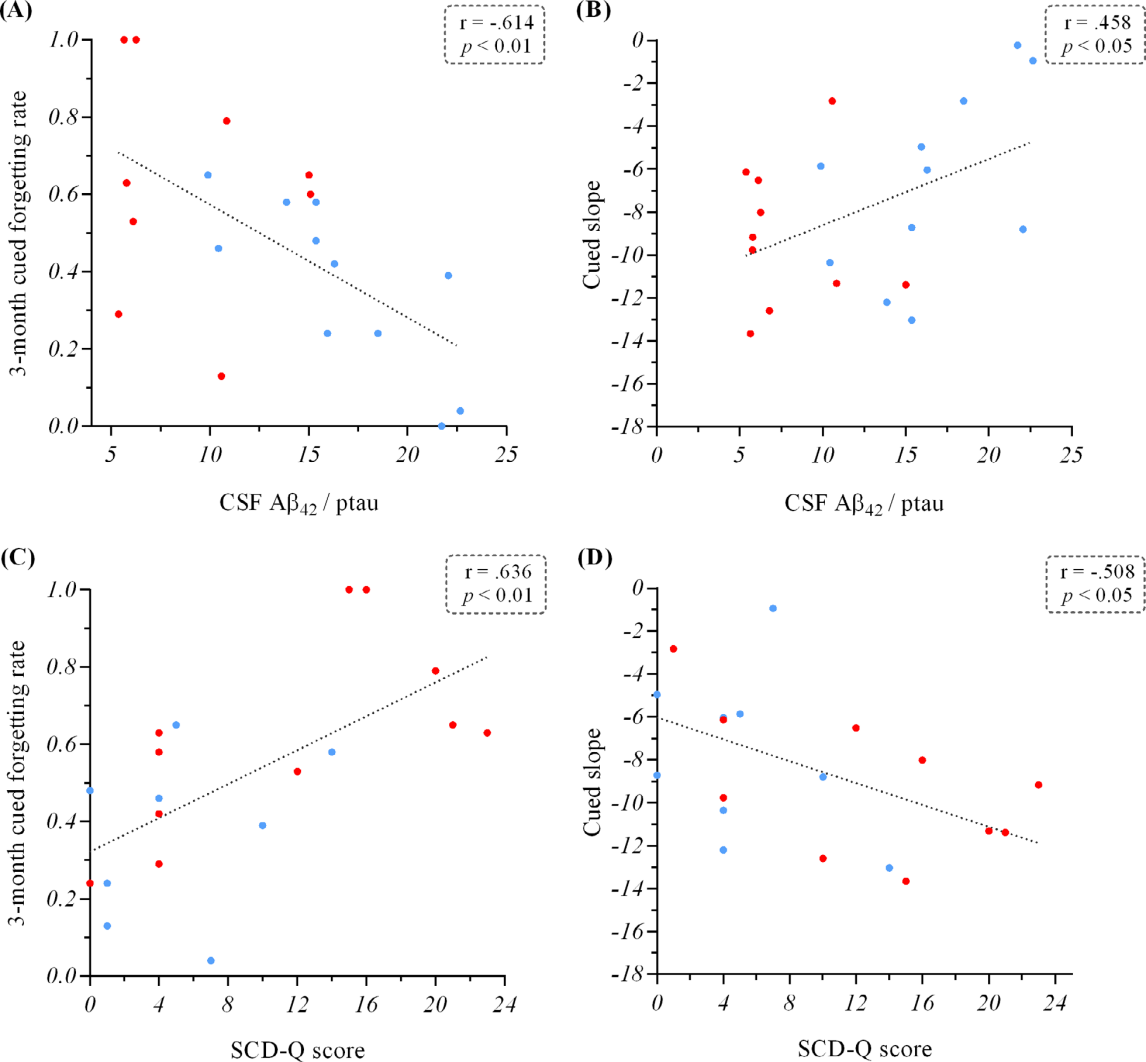
Panels A and B show the correlation between the ratio CSF Aβ_42_/ phosphorylated tau and the 3‐month cued forgetting rate and the cued slope from the AFE‐T, respectively. Panels C and D show the correlations between the SCD‐Q score and the 3‐month cued forgetting rate and the cued slope, respectively. Key: Blue dots, APOE ɛ4 noncarriers; Red dots, APOE ɛ4 carriers; CSF, cerebrospinal fluid; Aβ_42_, Amyloid‐β isoform 42; ptau, CSF phosphorylated tau levels; SCD‐Q, Subjective Cognitive Decline Questionnaire.

Correlational analyses in the whole sample showed a significant negative correlation between the 3‐month cued forgetting rate and the ratio CSF Aβ_42_/ptau (r = −.614; *P *< 0.01; Figure 2A). This correlation remained significant after controlling for potential confounders such as age (r = −.481; *P *< 0.05), years of education (r = −.606; *P *< 0.01) or the delay between CSF AD biomarkers acquisition and AFE‐T testing (r = −.548; *P *< 0.05). Besides, a significant positive correlation between the *cued slope* (from the modeled function in each individual) and the ratio Aβ_42_/ptau (r = .458; *P *< 0.05; Figure 2B) was found, again indicating that higher forgetting was associated to lower (more pathologic) levels of CSF AD biomarkers.

A significant positive correlation was found between the 3‐month cued forgetting rate and the SCD‐Q score (r = .636; *P *< 0.01; Figure 2C), and a significant negative correlation was found between the *cued slope* and the SCD‐Q (r = −.508; *P *< 0.05; Figure 2D), showing that higher forgetting was associated with higher cognitive concerns.

## Discussion

Using a new highly demanding associative memory test, AFE‐T, we examined ALF in cognitively healthy APOE ɛ4 carriers and its relationships with AD CSF biomarkers. The results revealed ALF in cued recall over three months in our well‐characterized sample of APOE ε3/ε4 heterozygotes. Moreover this performance was positively associated with core AD CSF biomarkers and subjective cognitive scores.

The effects of APOE ε4 on memory performance in clinically normal adults and its relationship with amyloid burden have been widely studied.^11^, ^12^, ^13^ As expected, our results showed that APOE ε4 carriers presented lower CSF Aβ levels than noncarriers (Table 1). However, data on the link between ALF and AD is still limited. Recent evidence from animal research showed accelerated forgetting with intact learning performance in a study using a model of familial AD (pre‐pathological PDAPP mice).^14^ In humans, ALF has been proposed as a potential cognitive marker for asymptomatic stages of AD. In an earlier report, our group provided evidence for subtle learning dysfunction and long‐term forgetting in preclinical AD subjects by employing the AFE‐T.^2^ A more recent study on a cohort of autosomal dominant AD families showed one‐week ALF in presymptomatic mutation carriers by using three standard cognitive tests^1^. Moreover Zimmerman & Butler^3^ revealed ALF in asymptomatic APOE ɛ4 carriers by assessing 60 participants (20 homozygous for ɛ3, 20 heterozygous for ɛ3 and ɛ4, and 20 homozygous for ɛ4) with a standard memory test. Participants were asked to learn a 15‐word list from the Rey Auditory Verbal Learning Test (RAVLT)^15^ on four consecutive trials to an 80% accuracy criterion. In their analyses, APOE status had no effects on memory encoding or short‐term recall, but was associated with long‐term forgetting over one week.

In line with Zimmermann & Butler,^3^ we found intact learning and ALF in APOE ε4 carriers. However, here we employed AFE‐T in order to explore participants’ cognition in a more comprehensive way. AFE‐T requires forming new associations or binding information without previous semantic knowledge, which is setting high demands on cognitive processing,^16^ especially compared to other standard episodic memory tasks. Regarding the learning performance, AFE‐T allowed a deeper analysis of encoding processes by yielding free and cued recall scores. Another strength is that we did not predefine any learning accuracy criterion which limits scoring range and thereby the chances for finding between‐group differences. The setup with AFE‐T also allowed exploration of free and cued recall for a longer time period, tracking ALF over 6 months after the learning phase. While the one‐week session appeared to be too close to initial learning, the 3‐month session was the optimal time‐point to find ALF in our APOE ε4 carriers, and cued recall measures showed higher sensitivity than spontaneous naming. Although the APOE ε4 carriers also performed worse than noncarriers on the free recall, the lack of statistically significant between‐group differences in these measures might be related to task difficulty and the floor effects observed beyond the 1‐week session. Also, the lack of significant between‐group differences in the 6‐month session might be due to the missing data in the APOE carriers group, which would also explain the slight improvement of this group compared to its performance at 3 months.

Importantly, both groups fitted the logarithmic function nearly perfect and particularly for cued recall, the APOE ε4 carriers showed a steeper slope (comprehensive forgetting measure) compared to noncarriers, indicating an overall tendency to show larger information loss after the initial learning in the carrier group, especially in between the first week and the 3‐month period. It is important to note that the slope parameter from the employed function strongly correlated with the AFE‐T forgetting rate at 3 months, demonstrating that both measures are clearly associated and again pointing out to the idea that the information is lost to a larger extent during the initial period after encoding. These results may also explain why the correlations found are larger with the 3‐month forgetting rate and the slope. After three months the rate of information loss is very small and therefore it might be futile to evaluate ALF beyond this time point. Taken together, our findings speak for the use of more sensitive measures with longer follow‐up designs when studying cognitively healthy at‐risk subjects. ALF is emerging as a cognitive feature of presymptomatic AD, highlighting the utility of long‐term recall designs on monitoring cognitive changes in this population.

Similar to the previous studies,^1^, ^3^ we found a relationship between self‐perceived subjective cognitive decline and ALF. This is an important finding since subjective cognitive concerns have been suggested as a risk factor for cognitive decline and dementia.^17^, ^18^ Furthermore, the present results showed a strong correlation between ALF (measured by cued forgetting rates and the slope parameter from the AFE‐T) and the CSF Aβ_42_/ptau ratio. Importantly, CSF Aβ_42_ and CSF ptau levels are valid indicators of the abnormal protein deposits underlying AD pathophysiology (i.e., β‐amyloid plaques and neurofibrillary tangles, respectively) and define AD as a specific neurodegenerative disease amongst other cognitive disorders.^19^ Our finding on the correlation between ALF as measured by AFE‐T and the ratio CSF Aβ_42_/ptau provides further evidence for ALF as a promising candidate for a specific marker of AD‐related subtle cognitive decline in presymptomatic stages of the disease. Finally, it is important to note that given our relatively small sample size, validation of the present results is called for in future studies including larger samples and more robust power analyses.

In sum, ALF is detectable in at‐risk individuals, and there is a relationship between this cognitive measure and the pathophysiological processes underlying AD. We strongly recommend the use of more demanding cognitive tests including long‐term forgetting measures for identifying and tracking the earliest cognitive manifestations in presymptomatic AD. The present results show that accelerated forgetting is able to capture cognitive dysfunction earlier in the AD continuum. Thus, they are particularly relevant for AD prevention trials, in which regulatory agencies require a change in cognition/functional status and not only a biomarker change.

## Author Contributions

A.R.F, L.R., M.L., and A.T.M. contributed to the conception and design of the study. All authors contributed to acquisition and analysis of the data. A.T.M., A.R.F., M.L., and L.R. contributed to drafting a significant portion of the manuscript. All authors have reviewed the manuscript and approved the final version.

## Conflicts of Interest

Nothing to report.
